# Phase prediction, microstructure, and mechanical properties of spark plasma sintered Ni–Al–Ti–Mn–Co–Fe–Cr high entropy alloys

**DOI:** 10.1186/s11671-023-03889-3

**Published:** 2023-09-19

**Authors:** Emmanuel Olorundaisi, Bukola J. Babalola, Moipone L. Teffo, Ufoma S. Anamu, Peter A. Olubambi, Juwon Fayomi, Anthony O. Ogunmefun

**Affiliations:** 1 Centre for Nanoengineering and Advanced Materials, School of Mining, Metallurgy and Chemical Engineering, University of Johannesburg, Johannesburg, 2092 South Africa; 2 Department of Chemical, Metallurgical and Materials Engineering, Institute for Nanoengineering Research, Tshwane University of Technology, Pretoria, South Africa; 3 Center for Additive Manufacturing, School of Engineering, RMIT University, Melbourne, 3000 Australia

**Keywords:** Nickel aluminide, High entropy alloy, Phase formation, Crystal structure, Nanoindentation, Microstructure

## Abstract

The effect of mechanical alloying on the development of Ni–Al–Ti–Mn–Co–Fe–Cr high entropy alloys (HEAs) utilizing the spark plasma sintering (SPS) method is the main goal of this study. A bulk sample was fabricated using SPS after the alloys were mixed for 12 h. Thermodynamic simulation, X-ray diffraction, scanning electron microscopy, nanoindentation, and microhardness were used to investigate the microstructure and mechanical properties of the as-mixed powders. The master alloy was made of NiAl and was subsequently alloyed with Ti, Mn, Co, Fe, and Cr at different compositions to develop HEAs at a sintering temperature of 850 °C, a heating rate of 100 °C/min, a pressure of 50 MPa, and a dwelling time of 5 min. A uniform dispersion of the alloying material can be seen in the microstructure of the sintered HEAs with different weight elements. The grain size analysis shows that the Ni_25_Al_25_Ti_8_Mn_8_Co_15_Fe_14_Cr_5_ alloy exhibited a refined structure with a grain size of 2.36 ± 0.27 µm compared to a coarser grain size of 8.26 ± 0.43 μm attained by the NiAl master alloy. Similarly, the HEAs with the highest alloying content had a greater microstrain value of 0.0449 ± 0.0036, whereas the unalloyed NiAl had 0.00187 ± 0.0005. Maximum microhardness of 139 ± 0.8 HV, nanohardness of 18.8 ± 0.36 GPa, elastic modulus of 207.5 ± 1.65 GPa, elastic recovery (*W*_e_/*W*_t_) of 0.556 ± 0.035, elastic strain to failure (*H*/*E*_r_) of 0.09.06 ± 0.0027, yield pressure (*H*^3^/$E_{{\text{r}}}^{2}$) of 0.154 ± 0.0055 GPa, and the least plasticity index (*W*_p_/*W*_t_) of 0.444 ± 0.039 were attained by Ni_25_Al_25_Ti_8_Mn_8_Co_15_Fe_14_Cr_5_. A steady movement to the left may be seen in the load–displacement curve. Increased resistance to indentation by the developed HEAs was made possible by the increase in alloying metals, which ultimately led to higher nanohardness and elastic modulus.

## Introduction

The reduction in carbon emissions is a major factor in the actualization of 4^th^ industrial revolution. This has necessitated an increasing demand in structural lightweight materials. There is a rising need for a material with a high strength-to-weight ratio and superior resistance to wear, corrosion, and creep. Transportation and chemical processing sectors need materials that can sustain stress and are fully dense, high strength, and tough in high-temperature applications. To achieve this need, a number of superalloys have been used; however, they have limitations due to their high-density properties, which necessitate significant mechanical loads to function [1, 2]. As a result, fuel use and carbon emissions into the atmosphere grow. The advent of nickel aluminide (NiAl) is a prospective material capable of replacing these alloys.

Nickel aluminide melts at a temperature as high as 1638 °C, exhibiting low-density properties and outstanding thermal and mechanical stability at high temperatures. This has placed nickel aluminide at an advantageous position for the development of alloys with a lower weight to a higher strength that has the benefit of having a stronger second phase for use in applications requiring high temperatures. With the presence of strong bond between nickel and aluminum at an elevated temperature, nickel aluminide has produced materials that are strong enough to compete with superalloys and ceramics and exhibit exceptional characteristics at high temperatures. Comparing the NiAl-based alloy to the traditional nickel-based superalloys, the former has demonstrated improved thermomechanical characteristics, outstanding corrosion, wear, and creep resistance [1–6]. Recent study has focused on the intermetallic compound nickel aluminide, which contains both aluminum and nickel. The two most widely known are Ni_3_Al and NiAl. But in this investigation, we'll concentrate on the NiAl.

At high temperatures, NiAl is renowned for its strength and brittleness as well as for having outstanding mechanical properties [7–9]. Often time, they show outstanding magnetic, superconducting, and chemical properties because of strong internal order and mixed bonding [10]. Given its widespread adoption, numerous experiments have been done to enhance its mechanical qualities using microalloying and fabrication techniques [11, 12]. As a result, it is crucial to develop a novel strategy for improving NiAl alloy by alloying with additional elements. Consequently, the addition of very stable metals to create high entropy alloy (HEA) gave NiAl alloy a tremendous strength at room temperature and higher temperatures, enhancing the strength and tribological properties. The HEAs are formed by the mixture of various fundamental elements in an equal or nearly equimolar ratios for the production of a multi-component alloys as oppose to the traditional alloying of a single-based element or compound to form the matrix [13].

HEA is a novel class of alloys that perform well mechanically at high temperatures [13]. Its distinctive properties thus increasing its developmental potential [14]. The interest in HEA from various sectors has increased dramatically during the past 16 years. Tsai [15] and Zhang et al. [16] have performed research on the material's physical attributes, such as its magnetic, electrical, or thermal qualities. Researchers have looked into HEAs exceptional mechanical, corrosive, and deformation behavior. This has broadened its use at extremely high and extremely low temperatures [17–25].

Developing NiAl-based HEA will be an innovative feat in solving most of the limitations associated with NiAl. However, the material's properties must be carefully taken into account for producing this NiAl-based HEA. The nature or type of the constituent elements, the alloys' weight % composition, the particle size, the cohesiveness of the continuous–discontinuous material interface, and the processing method used to alter the microstructural evolution are important [26]. HEAs structures are either FCC, BCC, HCP, or a mixture of any of the structures. The structure of HEA can be predicted in so many ways; one of the most used is the valence electron concentration (VEC). It is suitable for predicting HEA structural stability, which is closely related to the electron concentration (Hume–Rothery rules). From the investigation of Michael et al., it was established that the value of VEC determines the HEA structure [27]. FCC is formed when VEC > 8, when it is 6.87 ≤ VEC ≤ 8, a mixture of BCC and FCC is formed; however, when it is < 8, the material formed is BCC.

To achieve FCC solid solutions, stable elements such as Co, Cu, Mn, Fe, and Ni must be considered, while attaining BCC solid solutions, BCC stabilizers elements such as V, W, Cr, Al, and Ti must be given due consideration [28, 29]. It is important to develop a dual-phase HEA in order to attain high strength and flexibility [30, 31]. The BCC phase provides the strength of the material required while the FCC phase provides material flexibility [32–36]. Selecting the right process and materials is crucial for producing NiAl-based HEA. HEAs can be fabricated by melting, gasification, electrochemical, or solid state [36–42]. The most widely used technique is casting; however, it is limited by structural defects such as porosity caused by thermal expansion and contraction [36, 41]. Hence, microstructural homogeneity cannot be achieved. Processing rout of casting differs from that of sintering, which influences phase formation [43–45]. Intermetallic phases can also be formed in a sintered sample, just as seen in casting. In a sintering operation, plasma generated between the surfaces of particles under pressure and heat leads to the formation of bonding between the particles and, consequently, the formation of phases in the material. Even though the sintering technique attains consolidation of powders without attaining the liquid state (just as in casting), the reactivity between the particles under heat and pressure led to phase formation in the alloy. Relatively for the purpose of this research, no study was carried out to compare phase formation in casting and that of sintering. There is a parametric approach using physiochemical parameters such as enthalpy of mixing, entropy of mixing, melting points, atomic size difference, and valence electron concentration to define phase formation rules for HEAs [46, 47].

The work of Malihe Zeraati et al. established the possibility of developing a stable solid solution HEA of either FCC or BCC phase, in the amorphous phase and crystalline state using SPS [36]. In both situations, the stability is determined by the mixing entropy [36]. Thus, understanding the enthalpy and entropy of mixing is crucial due to the complexity of the alloying elements [47]. With the studies of some researchers, three rules on which the formation of solid solutions was established, namely: the enthalpy of mixing (Δ*H*_mix_), the entropy of mixing (Δ*S*_mix_), and the atomic size (*δ*) [46, 48–51]. In his work, Torralba et al. [47] emphasize the restrictions on these factors that encourage the emergence of basic phases. Such that: Δ*H*_mix_ value should not be too high due to big positive Δ*H*_mix_ causes phase separation and substantial negative Δ*H*_mix_ usually results in intermetallic phases. δ should be insignificantly enough given that a large δ causes unstable basic structures and produces unnecessary strain energy. The relationship between structure, VEC, Δ*H*_mix_, Δ*S*_mix_, *δ*, as well as the electronegativity difference, was critically discussed by Guo et al. [46]. Multi-component alloys were divided into amorphous phase, solid solution, and intermetallic compound in his study. Solid solution formation was discovered to be − 22 ≤ Δ*H*_mix_ ≤ 7 kJ/mol, 0 ≤ *δ* ≤ 8.5%, and 11 ≤ Δ*S*_mix_ ≤ 19.5 J/(K mol) [48]. Yang et al. [52] proposed an estimated solid solution formation parameter Ω, defined as *T*_m_Δ*S*_mix_/$\left| {\Delta H_{{{\text{mix}}}} } \right|$ to be ≥ 1.1, where T_m_ is given as the mole averaged melting point. By calculating the parameters of δ and Ω for typical multi-component, he also proposed δ ≤ 6.6% as a criterion for forming high entropy stabilized solid solution phase [52].

The ideal consolidation technique is spark plasma sintering (SPS), and the mechanical alloy procedure will result in fully pre-alloyed NiAl-based HEA powders [30, 32, 36, 40]. Due to the difficulty in producing complicated shapes, the SPS procedures have unquestionably been shown to be an excellent powder metallurgy (PM) strategy to generating nanostructured ultrafine-grained materials. It produces extremely dense, exceptionally well-bonded solid materials that resist grain coarsening [53]. By using this method, solid bulk materials might be better consolidated at low temperatures. It made it possible to incorporate a significant amount of particles that are often impossible to manufacture using other processing techniques [30, 32, 36, 40].

In the current study, NiAl alloys containing Ti, Mn, Fe, Co, and Cr were produced using the SPS method. It is impossible to overstate the impact of these alloying components. These alloying components are used to enhance the fabricated HEAs mechanical characteristics [54, 55]. The presence of Cr, Al, and Ti stabilized the BCC phase while Co, Mn, Fe, and Ni introduced the FCC phase [28, 29]. It is important to keep in mind that the alloying components have an impact on the ultimate properties of the fabricated HEA, including the melting temperature, density, and lattice constant. Hence, these alloys usually have better mechanical properties because a solid solution forms during their synthesis. Therefore, this research seeks to fabricate Ni–Al–Ti–Mn–Co–Fe–Cr HEA at varying weight constituents using spark plasma sintering (SPS) process. Also, the mechanical and microstructural behavior of the sintered Ni–Al–Ti–Mn–Co–Fe–Cr HEA will be examined.

## Materials and methods

### Thermodynamic simulation

The amount of phase and phase formation was determined using THERMOCALC software version 2021b with the TCHEA5 HEAs database. During the thermodynamic simulation, the solidus and liquidus temperature of the developed HEAs was determined. This result served as a guide during the sintering process. The empirical correlations between the physical parameters for the designing of HEA are theoretically analyzed below [46, 48, 49, 52]:

The mixing entropy (Δ*S*_mix_) is expressed as:

1 $$\Delta S_{{{\text{mix}}}} = - R\mathop \sum \limits_{i}^{n} C_{i} \ln C_{i}$$

where *R* = gas constant given as 8.314 J/(K mol), *C*_*i*_ atomic percent of the *i*th component. *n* number of alloy components.

The mixing enthalpy (Δ*H*_mix_) is estimated as:

2 $$\Delta H_{{{\text{mix}}}} = \mathop \sum \limits_{i = 1, j \ne }^{n} \Omega_{ij} C_{i} C_{j}$$

where Ω_ij_ = 4Δ*H*_AB_, Δ*H*_AB_ is expressed as the mixing enthalpy for binary alloys values from the Takeuchi et al. [56] table, based on the Miedema macroscopic model.

VEC is defined as:

3 $${\text{VEC}} = \mathop \sum \limits_{i}^{n} C_{i} \left( {{\text{VEC}}} \right)_{i}$$

$\left( {{\text{VEC}}} \right)_{i}$ is expressed as the valence electron concentration of the *i*th alloy element.

The atomic size difference (*d*) is defined conventionally as:

4 $$\delta = 100\sqrt {\mathop \sum \limits_{i = 1}^{n} C_{i} \left( {1 - \frac{{r_{i} }}{{\overline{r}}}} \right)^{2} }$$

where $\overline{r} = \mathop \sum \limits_{i = 1}^{n} C_{i} r_{i}$, $C_{i}$ = atomic percentage and $r_{i}$ = atomic radius of the individual alloy component.

### Experimental materials

Thermo Fisher Scientific supplied the powders used in this research, with details shown in Table 1.

**Table 1:** Details of starting powders

Elements	% purity	Particle size (μm)
Al	99.8	< 25
Ni	99.8	< 25
Ti	99.6	< 25
Mn	99.6	< 10
Co	99.5	< 37
Fe	99.6	< 15
Cr	99.2	< 10

### Fabrication of Ni–Al–Ti–Mn–Co–Fe–Cr high entropy alloy

In this research, NiAl was employed as the master alloy in the development of high entropy alloy (HEA). While Ti, Mn, Co, Fe, and Cr serve as the constituent alloying elements. The Ni–Al–Ti–Mn–Co–Fe–Cr powders were mixed at varying constituent weight ratio as presented in Table 2. The measured powder was placed in an airtight container and mixed at 150 rpm with a tubular mixer over the course of 12 h to further homogenize the alloy. A SPS machine, model SPS FCT Systeme GmbH, Germany, was used to consolidate the combined powder. This was carried out under constant sintering conditions of 850 °C for the temperature (ST), 50 MPa for the pressure (P), 100 °C/min for the heating rate (HR), and 5 min for the dwell time (DT). With the proper punches, the measured admixed metal alloys were poured into a 20-mm graphite die. A graphite sheet was used to create space between the powder and inside surface of the graphite die before adding powder. This was done in order to reduce the temperature differential on the workpiece and to facilitate the removal of the solidified powder after sintering. The sintered samples were then removed from the graphite die, cleaned of contaminants by sandblasting, grinded with different emery paper grades, and polished with the appropriate lubricants as part of the metallographic process.

**Table 2:** Compositions of the elements of the alloys

Elements	Al	Ni	Ti	Mn	Co	Fe	Cr
Sample A (at. %)	50	50	–	–	–	–	–
Sample A (wt. %)	31.493	68.507	–	–	–	–	–
Sample B (at. %)	14.286	14.286	14.286	14.286	14.286	14.286	14.286
Sample B (wt. %)	7.59	16.52	13.48	15.47	16.59	15.72	14.64
Sample C (at. %)	25	25	8	8	14	15	5
Sample C (wt. %)	13.79	30.00	7.83	8.99	18.08	15.99	5.32
Sample D (at. %)	25	25	8	8	15	14	5
Sample D (wt. %)	13.80	30.02	7.84	8.99	17.14	16.88	5.32
Sample E (at. %)	25	25	8	9	14	14	5
Sample E (wt. %)	13.80	30.03	7.84	10.12	16.89	16.00	5.32
Sample F (at. %)	25	25	9	8	14	14	5
Sample F (wt. %)	13.82	30.07	8.83	9.01	16.91	16.03	5.33

### Characterization and analysis of the sintered samples

#### Density analysis

The sintered HEAs experimental density was calculated by employing the Archimedes principles using a densitometer while the theoretical density was examined using the mixture's rule as expressed in Eq. 5. The mean value, which indicates the mean experimental density, was calculated from five measurements. Equations 6 and 7 were used to calculate the % porosity and relative density of the alloys based on experimental density.

5 $${\text{Theoritical}}\;{\text{density}} = \left( {\frac{{\% {\text{Ni}}}}{{\rho {\text{Ni}}}} + \frac{{\% {\text{Al}}}}{{\rho {\text{Al}}}} + \frac{{\% {\text{Ti}}}}{{\rho {\text{Ti}}}} + \frac{{\% {\text{Mn}}}}{{\rho {\text{Mn}}}} + \frac{{\% {\text{Fe}}}}{{\rho {\text{Fe}}}} + \frac{{\% {\text{Mn}}}}{{\rho {\text{Mn}}}} + \frac{{\% {\text{Cr}}}}{{\rho {\text{Cr}}}}} \right)^{ - 1}$$

6 $${\text{Relative}}\;{\text{density}} = \left( {\frac{{{\text{Experimental}}\;{\text{density}}}}{{{\text{Theoritical}}\;{\text{density}}}} \times 100} \right)\%$$

7 $${\text{Percentage}}\;{\text{Porosity}} = 100\% - {\text{Relative}}\;{\text{ density}}$$

#### Microstructure and crystalline phase characterization

Before using Keller's etchant, which includes 190 ml of distilled water, 5 ml of nitric acid, 3 ml of hydrochloric acid, and 2 ml of hydrofluoric acid to expose the grains, the samples were ground and polished. Using a JEOL JSM-7900F scanning electron microscopy, the surface microstructural of the developed HEAs was studied. The degree of the bonding structure, the grain patterns along the borders, and the grain size were all examined using SEM images. The Panalytical X'Pert Pro diffractometer was also employed to assess the X-ray diffraction (XRD) of the sintered sample. It operates at a 1.5406 A wavelength with a 5–90 degree angle range and a 40 kV, 20 mA Cu–K source. We looked into the samples' chemical composition and crystalline phase. Each sample's crystallite size at the nanoscale was calculated using the Scherrer formula, which is presented in Eq. 8. The microstrain was determined using the relationship in Eq. 9. Similarly, ImageJ software was used to determine the sintered HEAs grain size.

8 $$D = \frac{K\lambda }{{\beta \cos \theta }}$$

9 $$\varepsilon = \frac{\beta }{4\tan \theta }$$

The parameters are defined thus: *D* = size of the crystallites, *K* = 0.9, *λ* 0.154060 nm, *β* = FWHM in radians, *θ* = peak position in radians, and $\varepsilon$ = microstrain.

#### Microhardness

The microhardness behavior was examined using the diamond indenter equipped INNOVATEST FALCON 500. This was done with an applied load of 100 gf for 15-s DT at 0.5-mm intervals. Each sample was indented five times, and the average was recorded as hardness.

#### Nanoindentation and strength mechanism of the developed Ni–Al–Ti–Mn–Co–Fe–Cr HEA

Nanoindentation analysis was employed to determine the nanohardness and elastic modulus at room temperature using a nanoindenter (NHT^3^, Switzerland). This was achieved at a range of applied force of 0.1 mN–500 mN, applied load of 100 mN, and a loading and unloading rate of 10 mN/min. Each sample underwent the Oliver and Pharr technique analysis after 10 indentations [57]. Using this technique, the load–displacement curve was used to analyze the nanohardness (*H*_N_), reduced elastic modulus (*E*_r_), and elastic modulus (*E*):

10 $$H_{{\text{N}}} = \frac{{F_{\max } }}{{A_{{\text{c}}} }}$$

*F*_max_ = maximum applied force and *A*_c_ = contact area.

From Eq. 11, at F_max_, the h_max_ is obtained by adding h_c_ and h_e_.

11 $$h_{\max } = h_{{\text{e}}} + h_{{{\text{c}} }}$$

*h*_max_ = maximum displacement depth, *h*_c_ = contact depth, and *h*_e_ = displaced elastic surface.

The relationship between stiffness *S*, *E*_r_, and *A*_c_ is presented in Eq. 12.

12 $$S = \beta \frac{2}{\sqrt \pi }E_{{\text{r}}} \sqrt {A_{{\text{c}}} }$$

The *E*_r_ (Eq. 13) is a derivative of the Poisson’s ratio (*V*_i_) of indenter, nanoalloy specimen (*V*_s_), sample elasticity (*E*_s_), and indenter elastic modulus (*E*_i_) [57–61].

13 $$\frac{1}{{E_{{\text{r}}} }} = \frac{{1 - V_{{\text{s}}}^{2} }}{{E_{{\text{s}}} }} + \frac{{1 - V_{{\text{i}}}^{2} }}{{E_{{\text{i}}} }}$$

Based on the loading displacement curve as shown in Eq. 14, an estimate of the total work in terms of elastic and plastic energy was made.

14 $$W_{{\text{t}}} = W_{{\text{e}}} + W_{{\text{p}}}$$

*W*_e_ and *W*_p_ represent the elastic and plastic energies, respectively, and *W*_t_ is the total energy used during the nanoindentation of the load–displacement curve.

Additionally, the total energy from Eq. 15 was used to calculate the elastic recovery index, which specifies the amount of energy released by the developed HEAs under a precise applied load. The elastic recovery established the resistance of the sintered alloy to impact load [57–61].

15 $${\text{Elastic}}\; {\text{recovery}}\; {\text{index}} = \frac{{W_{{\text{e}}} }}{{W_{{\text{t}}} }}$$

Furthermore, the plasticity index is given in Eq. 16. This depicts the intrinsic plasticity of the fabricated HEA.

16 $${\text{Plasticity}}\;{\text{index}} = \frac{{W_{{\text{p}}} }}{{W_{{\text{t}}} }}$$

The reduced elastic modulus and nanohardness as described in Eqs. 17 and 18 are used to estimate the yield pressure and elastic strain, which represent the resistance of the manufactured HEA to plastic deformation and failure, respectively [57–61].

17 $${\text{Yield}}\;{\text{Pressure}} = \frac{{H^{3} }}{{E_{{\text{r}}}^{2 } }}$$

18 $${\text{Elastic}}\;{\text{strain}}\;{\text{to}}\;{\text{failure}} = \frac{H}{{E_{{\text{r}}} }}$$

## Results and discussion

### The microstructural characterization of the sintered Ni–Al–Ti–Mn–Co–Fe–Cr HEA

**Fig. 1:**
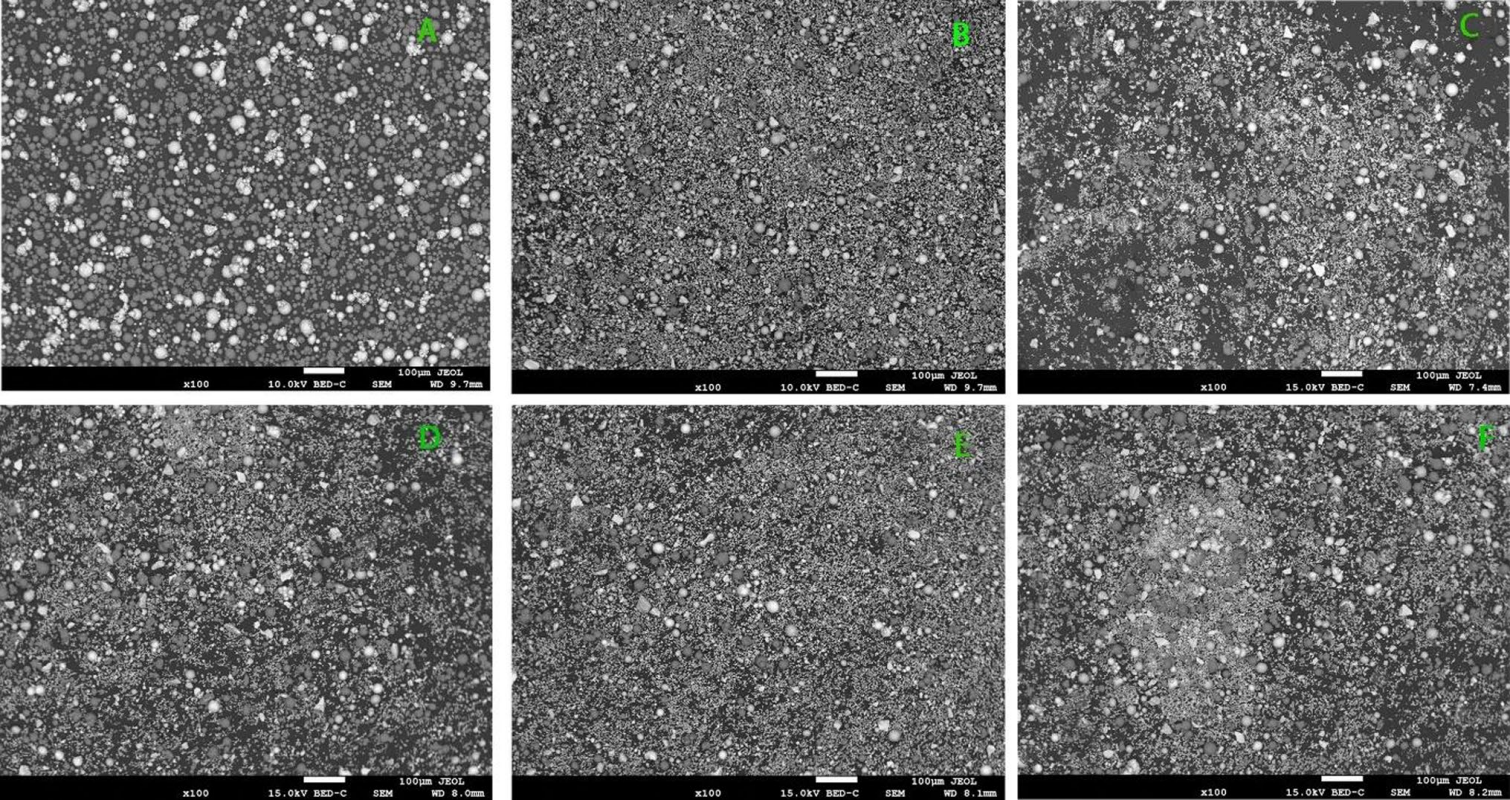
SEM of mixed Ni–Al–Ti–Mn–Co–Fe–Cr system: **A** Ni_50_Al_50_, **B** Ni_14.286_Al_14.286_Ti_14.286_Mn_14.286_Co_14.286_Fe_14.286_Cr_14.286_, **C** Ni_25_Al_25_Ti_8_Mn_8_Co_14_Fe_15_Cr_5_, **D** Ni_25_Al_25_Ti_8_Mn_8_Co_15_Fe_14_Cr_5_, **E** Ni_25_Al_25_Ti_8_Mn_9_Co_14_Fe_14_Cr_5_, and **F** Ni_25_Al_25_Ti_9_Mn_8_Co_14_Fe_14_Cr_5_

Figure 1 shows the homogeneity of the admixed powders before sintering. The SEM analysis reviews evenly dispersion of the alloying materials.

**Fig. 2:**
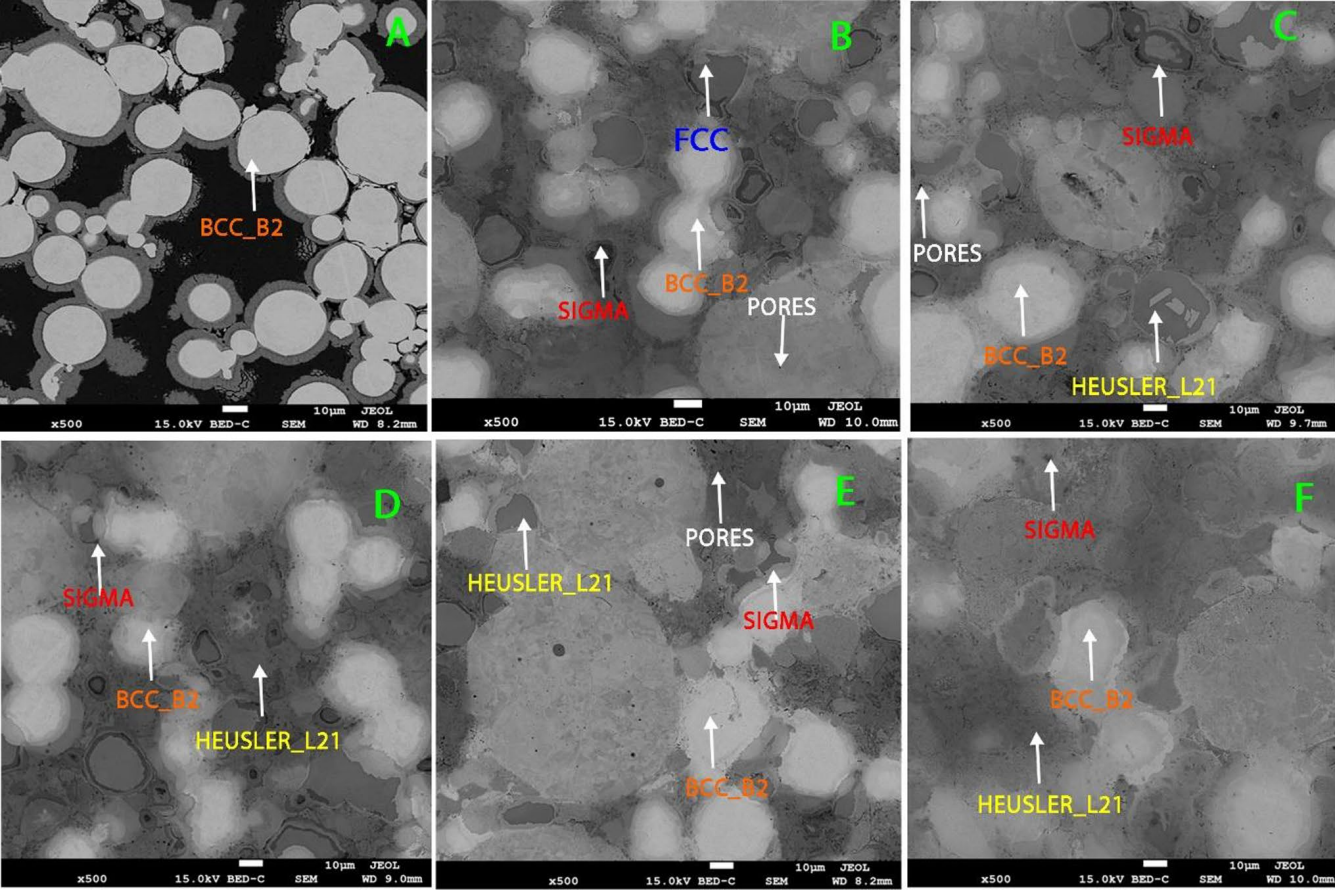
SEM of sintered Ni–Al–Ti–Mn–Co–Fe–Cr HEA at varying weight: **A** Ni_50_Al_50_, **B** Ni_14.286_Al_14.286_Ti_14.286_Mn_14.286_Co_14.286_Fe_14.286_Cr_14.286_, **C** Ni_25_Al_25_Ti_8_Mn_8_Co_14_Fe_15_Cr_5_, **D** Ni_25_Al_25_Ti_8_Mn_8_Co_15_Fe_14_Cr_5_, **E** Ni_25_Al_25_Ti_8_Mn_9_Co_14_Fe_14_Cr_5_, and **F** Ni_25_Al_25_Ti_9_Mn_8_Co_14_Fe_14_Cr_5_

The microstructural evolution of the sintered Ni–Al–Ti–Mn–Co–Fe–Cr HEA is shown in Fig. 2. The figure revealed microstructure obtained under different weight ratios but subjected to the same sintering conditions. The SEM result shows a well-refined grains with high necking between the particles, which improves stronger interparticle bonding and reduction of pores. The SEM result reveals the presence of four major phases which are dependent on the varying weight ratio. The microstructure, particularly at the grain boundaries, exhibited a homogeneous dispersion of alloying elements with no noticeable agglomeration. The prevention against the development of grain growth is created by the adhesive connection between the alloy's constituent alloying elements, which also enhances the nucleation of the phases [62]. As shown from Fig. 2A, the BCC phase is predominantly presence which is responsible for the brittleness of NiAl alloy at room temperature. To improve on this alloy, it necessitates the inclusion of other metallic alloys in different weight ratios as shown in Fig. 2B–F.

**Fig. 3:**
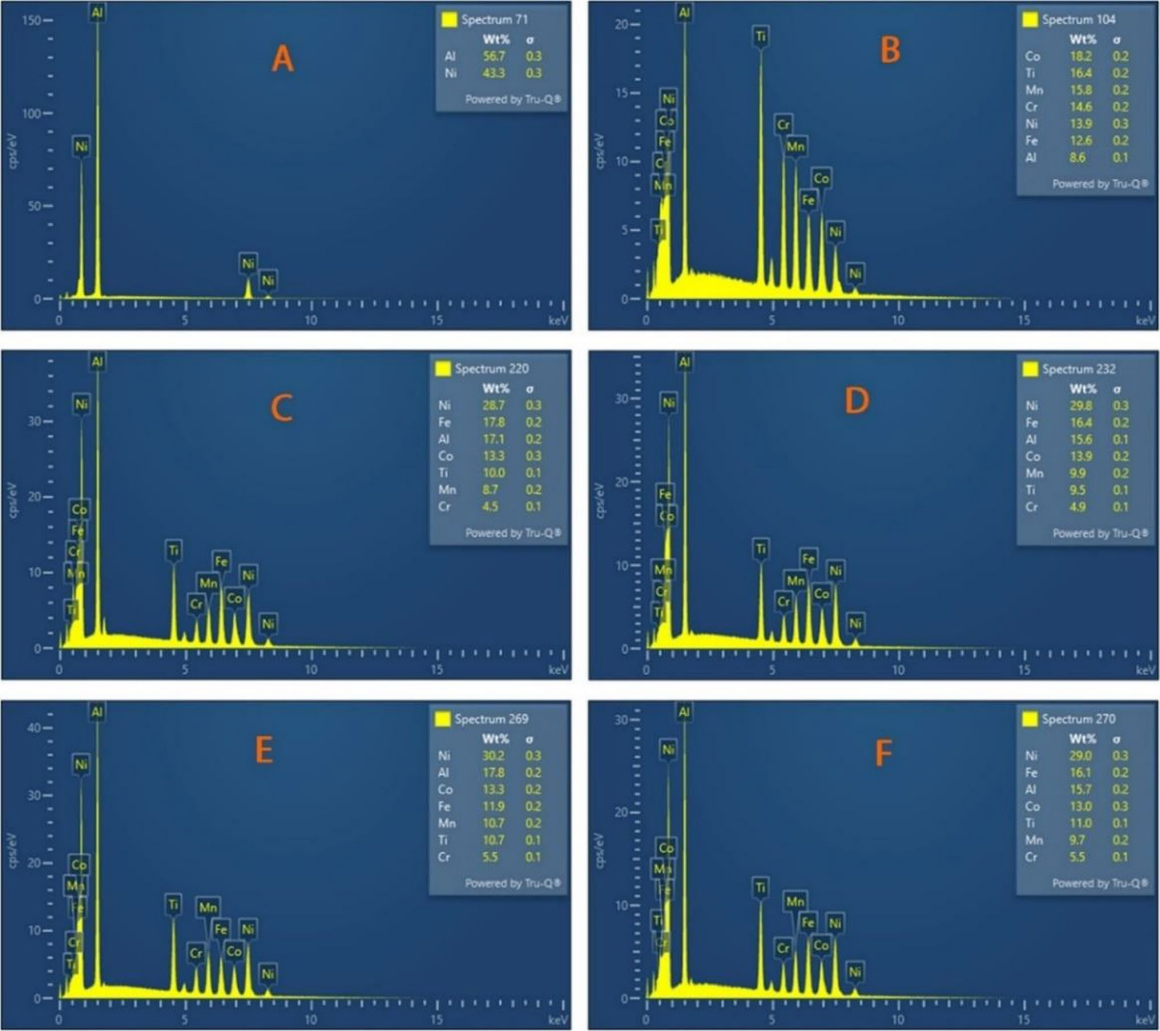
EDS analysis of sintered Ni–Al–Ti–Mn–Co–Fe–Cr system: **A** Ni_50_Al_50_, **B** Ni_14.286_Al_14.286_Ti_14.286_Mn_14.286_Co_14.286_Fe_14.286_Cr_14.286_, **C** Ni_25_Al_25_Ti_8_Mn_8_Co_14_Fe_15_Cr_5_, **D** Ni_25_Al_25_Ti_8_Mn_8_Co_15_Fe_14_Cr_5_, **E** Ni_25_Al_25_Ti_8_Mn_9_Co_14_Fe_14_Cr_5_, and **F** Ni_25_Al_25_Ti_9_Mn_8_Co_14_Fe_14_Cr_5_

Interestingly, the inclusion of Ni–Al–Ti–Mn–Co–Fe–Cr in equal atomic (Fig. 2B) introduces the FCC phases which improves theductility nature of the developed HEA. However, it was discovered that, as the weight percent was varied, the FCC phases disappear, and following the maximal nucleation of the FCC phase, significant grain refining to a smaller grain size was accomplished. A new phase is introduced in Fig. 2C–F known as the HEUSLER. The HEUSLER phase is a magnetic intermetallics with FCC crystal structure [63–65]. It is paramount to note that the even dispersion of the alloying materials has the tendency of enhancing the microstructural and mechanical behavior of the developed HEA, by attaining a strong bonding between the constituent elements without noticeable deficiencies such as cracks and pores [62] (Fig. 3).

**Fig. 4:**
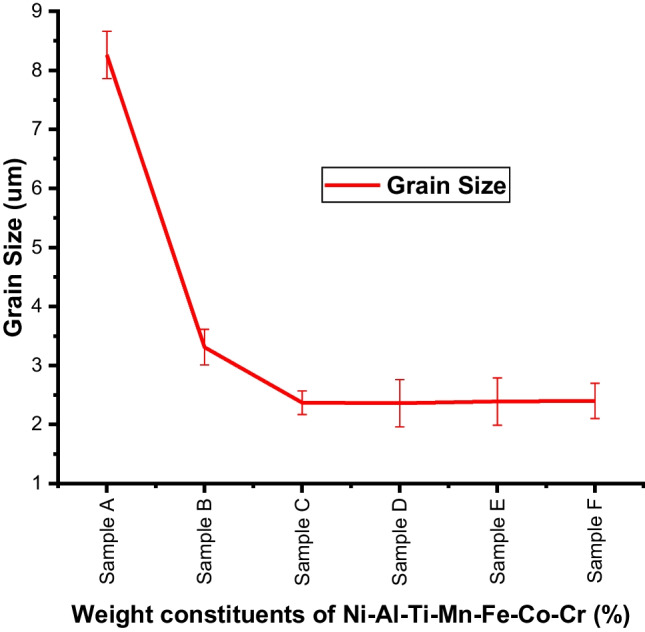
The grain size of the developed Ni–Al–Ti–Mn–Co–Fe–Cr system: **A** Ni_50_Al_50_, **B** Ni_14.286_Al_14.286_Ti_14.286_Mn_14.286_Co_14.286_Fe_14.286_Cr_14.286_, **C** Ni_25_Al_25_Ti_8_Mn_8_Co_14_Fe_15_Cr_5_, **D** Ni_25_Al_25_Ti_8_Mn_8_Co_15_Fe_14_Cr_5_, **E** Ni_25_Al_25_Ti_8_Mn_9_Co_14_Fe_14_Cr_5_, and **F** Ni_25_Al_25_Ti_9_Mn_8_Co_14_Fe_14_Cr_5_

The degree of grain refinement during the recrystallization process of the developed HEA was further studied by measuring the grain size using ImageJ software (Fig. 4). Table 3 shows the grain size of the fabricated HEA being influenced by the variations in alloying elements’ composition. Fundamentally, the observed decrease in grain size was impacted by the nano-weight inclusion's composition and particle size. However, the size for the non-equal atomic (samples C–F) has almost the same grain size, this is because the marginal differences in the compositions are close. The effect of mechanical alloying on reducing initial particle size and variation of the alloys' composition with elements having lower particle size contributes to the variations in grain size. The incorporation of alloying materials led to dynamic recrystallization and plastic deformation, which is best explained as the source of the refined grain structure and possibly precipitate dissolution during the sintering process. Recrystallization also prevents grain growth and guarantees that the refined grains are distributed uniformly [62, 66, 67]. The EDS analysis ascertains the chemical constituent of the sintered HEA (Fig. 3).

**Table 3:** Grain size of the manufactured Ni–Al–Ti–Mn–Co–Fe–Cr HEA

Sample (s)	Grain size (µm)
Ni_50_Al_50_	8.26 ± 0.43
Ni_14.286_Al_14.286_Ti_14.286_Mn_14.286_Co_14.286_Fe_14.286_Cr_14.286_	3.31 ± 0.34
Ni_25_Al_25_Ti_8_Mn_8_Co_14_Fe_15_Cr_5_	2.37 ± 0.22
Ni_25_Al_25_Ti_8_Mn_8_Co_15_Fe_14_Cr_5_	2.36 ± 0.41
Ni_25_Al_25_Ti_8_Mn_9_Co_14_Fe_14_Cr_5_	2.39 ± 0.36
Ni_25_Al_25_Ti_9_Mn_8_Co_14_Fe_14_Cr_5_	2.4 ± 0.27

### Phase analysis of the fabricated Ni–Al–Ti–Mn–Co–Fe–Cr HEA

**Fig. 5:**
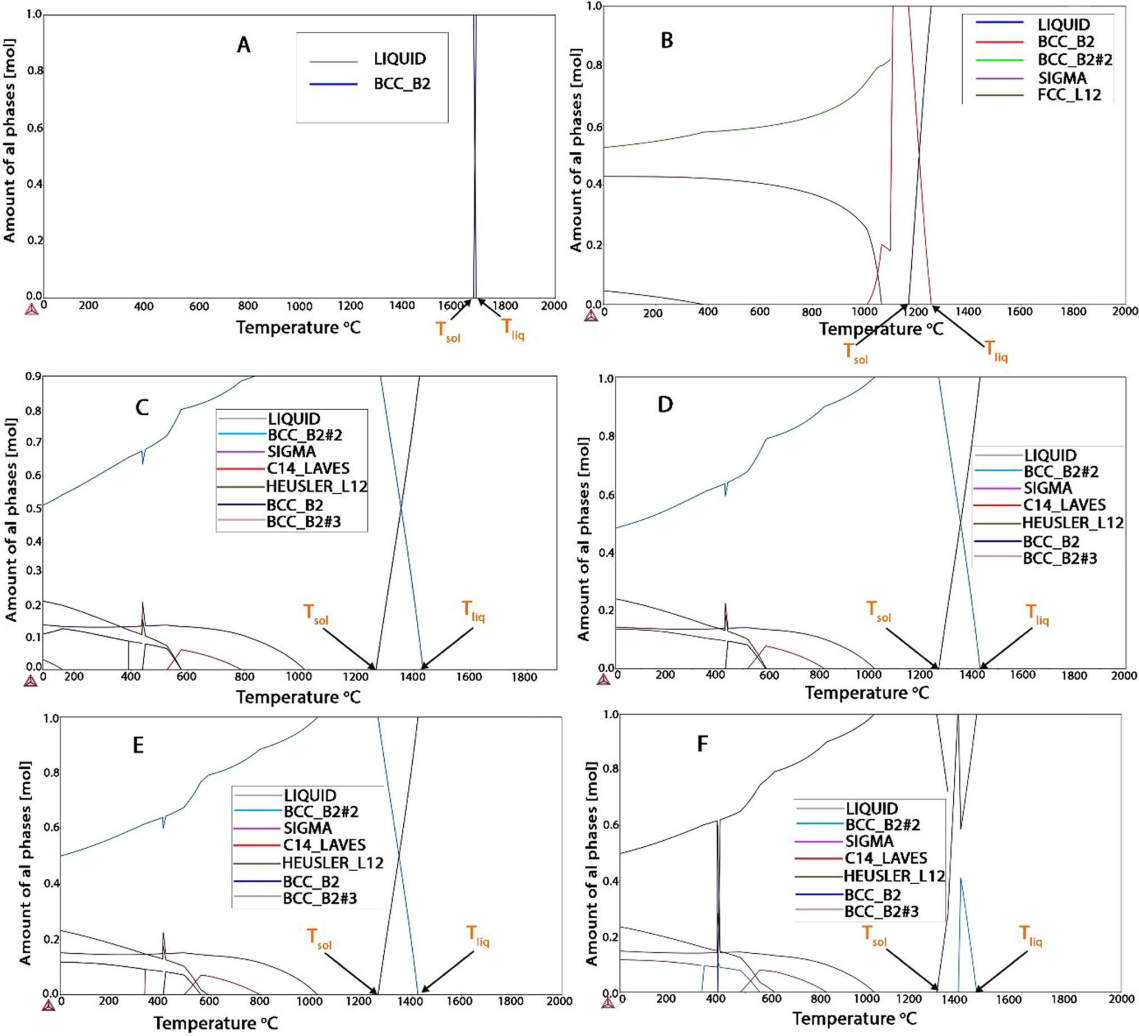
Ni–Al–Ti–Mn–Co–Fe–Cr amount of phase; **A** Ni_50_Al_50_, **B** Ni_14.286_Al_14.286_Ti_14.286_Mn_14.286_Co_14.286_Fe_14.286_Cr_14.286_, **C** Ni_25_Al_25_Ti_8_Mn_8_Co_14_Fe_15_Cr_5_, **D** Ni_25_Al_25_Ti_8_Mn_8_Co_15_Fe_14_Cr_5_, **E** Ni_25_Al_25_Ti_8_Mn_9_Co_14_Fe_14_Cr_5_, and **F** Ni_25_Al_25_Ti_9_Mn_8_Co_14_Fe_14_Cr_5_

**Fig. 6:**
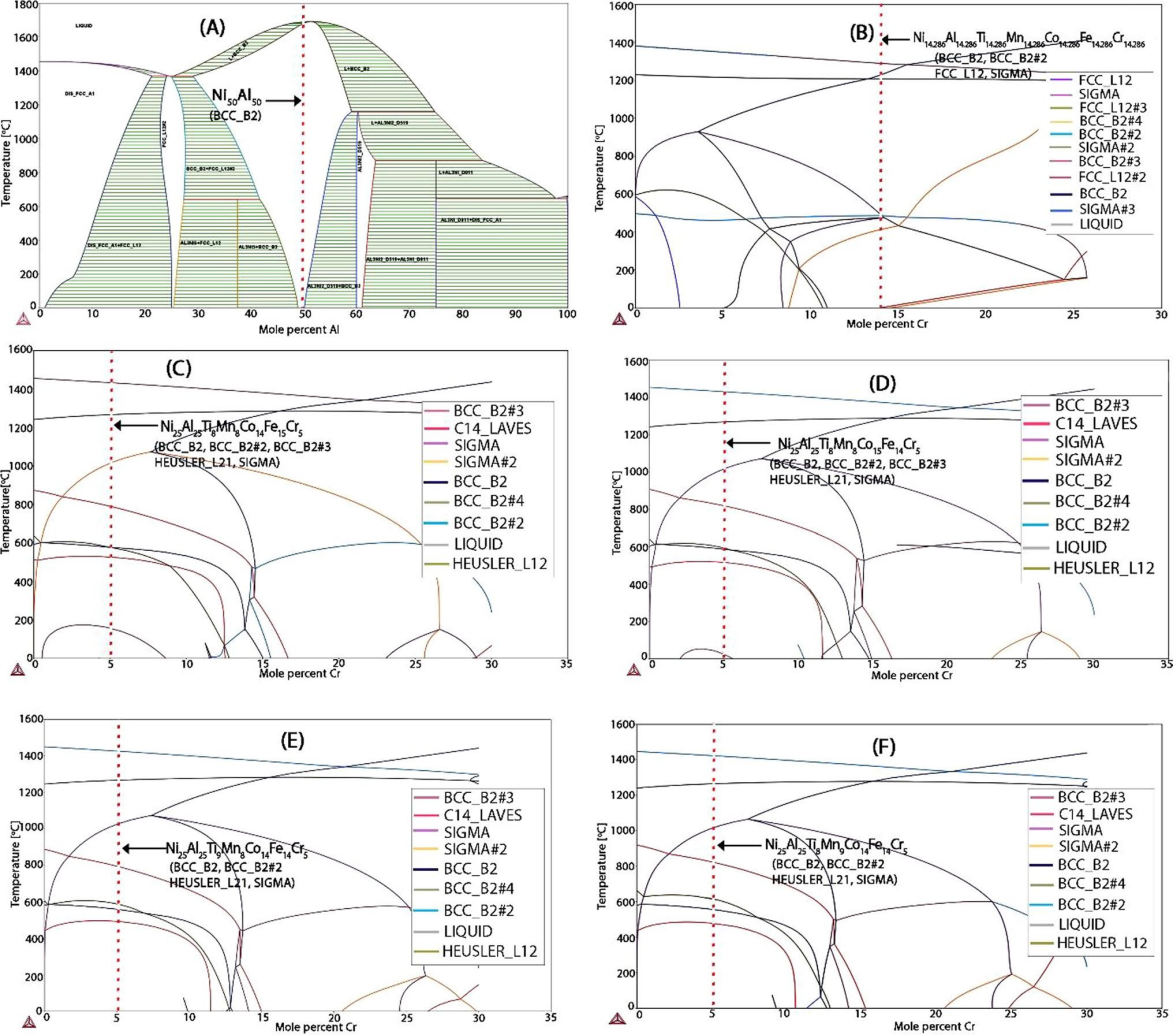
Ni–Al–Ti–Mn–Co–Fe–Cr phase diagram: **A** Ni_50_Al_50_, **B** Ni_14.286_Al_14.286_Ti_14.286_Mn_14.286_Co_14.286_Fe_14.286_Cr_14.286_, **C** Ni_25_Al_25_Ti_8_Mn_8_Co_14_Fe_15_Cr_5_, **D** Ni_25_Al_25_Ti_8_Mn_8_Co_15_Fe_14_Cr_5_, **E** Ni_25_Al_25_Ti_8_Mn_9_Co_14_Fe_14_Cr_5_, and **F** Ni_25_Al_25_Ti_9_Mn_8_Co_14_Fe_14_Cr_5_

**Fig. 7:**
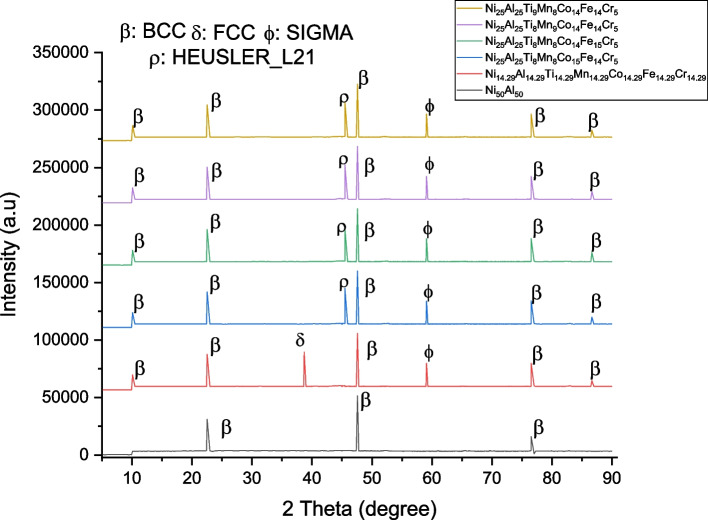
The XRD phases of the sintered samples

With the use of CALPHAD-based tools and THERMOCALC software, phase formation and identification were predicted. Figures 5 and 6, respectively, display the phase diagram and the amount of phase. Figures 5a and 6a represent the master alloy (NiAl), while 5b–f and 6b–f depict the developed HEA (Ni–Al–Ti–Mn–Fe–Co–Cr). Only the BCC phase is visible in NiAl. This phase is what causes NiAl to be so brittle [11]. FCC, HEUSLER, and SIGMA phases were added as a result of alloying of NiAl. The XRD finding in Fig. 7 provides additional experimental support for this conclusion and from the calculated VEC result. From the amount of phase diagram, the melting temperature of the developed alloy was predicted as shown from Fig. 5. However, the phase diagram successfully predicted the presence of each phase in the HEAs developed. The thermodynamic data obtained successfully predicted the solid solution formation. From Table 4, ΔS_mix_ and δ of the developed HEAs are in the range of the solid solution formation [68]. Similarly, VEC of Ni_14.286_Al_14.286_Ti_14.286_Mn_14.286_Co_14.286_Fe_14.286_Cr_14.286_, Ni_25_Al_25_Ti_8_Mn_8_Co_15_Fe_14_Cr_5_, and Ni_25_Al_25_Ti_8_Mn_9_Co_14_Fe_14_Cr_5_ falls into the range 6.87 ≤ VEC ≤ 8 which can be a sign of the FCC and BCC formation [68]. However, from the THERMOCALC result, only Ni_14.286_Al_14.286_Ti_14.286_Mn_14.286_Co_14.286_Fe_14.286_Cr_14.286_ has FCC present, this could be as a result of the VEC value of Ni_25_Al_25_Ti_8_Mn_8_Co_15_Fe_14_Cr_5_, and Ni_25_Al_25_Ti_8_Mn_9_Co_14_Fe_14_Cr_5_ being at the boundary; therefore, no or a negligible volume fraction of FCC is present. The thermodynamic information in Table 4 predicted the emergence of a solid solution phase with FCC and BCC structures.

**Table 4:** Ni–Al–Ti–Mn–Co–Fe–Cr alloy thermodynamic parameters

Sample	*δ*	Δ*S*_mix_ (KJ/mol)	VEC
Ni_50_Al_50_	1.26	5.76	6.5
Ni_14.286_Al_14.286_Ti_14.286_Mn_14.286_Co_14.286_Fe_14.286_Cr_14.286_	5.52	16.18	6.94
Ni_25_Al_25_Ti_8_Mn_8_Co_14_Fe_15_Cr_5_	5.19	15.02	6.71
Ni_25_Al_25_Ti_8_Mn_8_Co_15_Fe_14_Cr_5_	5.19	15.02	6.89
Ni_25_Al_25_Ti_8_Mn_9_Co_14_Fe_14_Cr_5_	5.31	15.07	6.88
Ni_25_Al_25_Ti_9_Mn_8_Co_14_Fe_14_Cr_5_	5.26	15.07	6.85

**Fig. 8:**
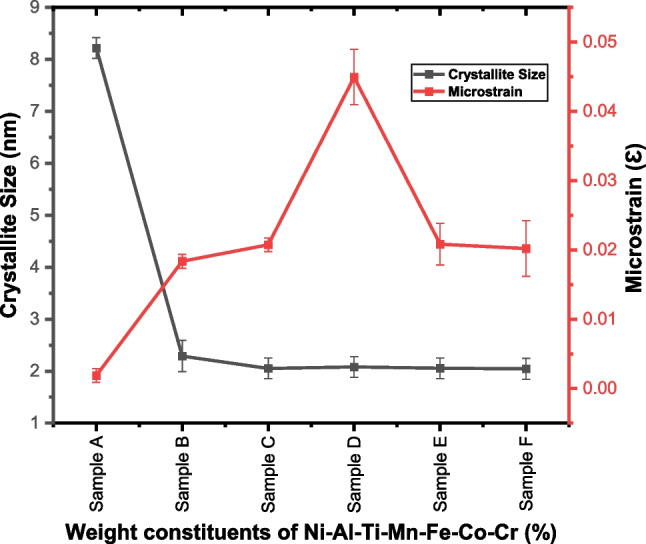
The crystallite size and microstrain of the sintered Ni–Al–Ti–Mn–Co–Fe–Cr system: **A** Ni_50_Al_50_, **B** Ni_14.286_Al_14.286_Ti_14.286_Mn_14.286_Co_14.286_Fe_14.286_Cr_14.286_, **C** Ni_25_Al_25_Ti_8_Mn_8_Co_14_Fe_15_Cr_5_, **D** Ni_25_Al_25_Ti_8_Mn_8_Co_15_Fe_14_Cr_5_, **E** Ni_25_Al_25_Ti_8_Mn_9_Co_14_Fe_14_Cr_5_, and **F** Ni_25_Al_25_Ti_9_Mn_8_Co_14_Fe_14_Cr_5_

The phase composition was examined by analyzing the XRD pattern. The strong bonding between the alloys is a reflection of the wettability between the NiAl and the alloying metals. The XRD and THERMOCALC results are in agreement. The BCC phase was consistently presence in the six samples. Ni_50_Al_50_ shows only the BCC phase, while Ni_14.286_Al_14.286_Ti_14.286_Mn_14.286_Co_14.286_Fe_14.286_Cr_14.286_ which is the equal atomic has three dominant phases BCC, FCC, and SIGMA. However, from Ni_25_Al_25_Ti_8_Mn_8_Co_14_Fe_15_Cr_5_, Ni_25_Al_25_Ti_8_Mn_8_Co_15_Fe_14_Cr_5_, Ni_25_Al_25_Ti_8_Mn_9_Co_14_Fe_14_Cr_5_, and Ni_25_Al_25_Ti_9_Mn_8_Co_14_Fe_14_Cr_5_, BCC, HEUSLER, and SIGMA phases were identified. From the diffractogram in Fig. 7, the BCC phase is more pronounced with a noticeable peak intensity and that indicates that precipitation from a solid solution has occurred [69–75]. A new peak was observed after the inclusion of equal atomic percent of the constituent alloys. The observed peak (FCC) provides proof that the Ni_50_Al_50_ was refined and dissolved through dynamic recrystallization. A further intriguing finding is the decrease in crystallite size with changing weight composition, as shown in Table 5. The degree of crystallization depends on the chemical compositions and thermal histories of the fabricated HEA [70, 73, 75]. The particle size of the fabricated HEA has a significant impact on both its physical and mechanical characteristics, which get better as size decreases [69]. The modifications in the grain structure that has been noticed are the result of these phases. For each of the fabricated HEA, the evaluated crystallinity size and lattice microstrain are shown in Fig. 8 based on the XRD patterns.

**Table 5:** The crystallite size of Ni–Al–Ti–Mn–Co–Fe–Cr HEA at different weight compositions

Sample	Crystallite size (nm)	Microstrain (*Ɛ*)
Ni_50_Al_50_	8.2183 ± 0.18	0.00187 ± 0.001
Ni_14.286_Al_14.286_Ti_14.286_Mn_14.286_Co_14.286_Fe_14.286_Cr_14.286_	2.2891 ± 0.31	0.0183 ± 0.001
Ni_25_Al_25_Ti_8_Mn_8_Co_14_Fe_15_Cr_5_	2.0527 ± 0.15	0.0207 ± 0.001
Ni_25_Al_25_Ti_8_Mn_8_Co_15_Fe_14_Cr_5_	2.081 ± 0.20	0.0449 ± 0.003
Ni_25_Al_25_Ti_8_Mn_9_Co_14_Fe_14_Cr_5_	2.0558 ± 0.16	0.0208 ± 0.003
Ni_25_Al_25_Ti_9_Mn_8_Co_14_Fe_14_Cr_5_	2.0448 ± 0.16	0.0201 ± 0.004

Compared to unalloyed NiAl, which has a crystallite size of 8.21 ± 0.81 nm, the developed HEAs crystallite size was well refined, according to the evaluated result. This is an indication of a unique influence of the microsized Ti–Mn–Co–Fe–Cr on the refinement of NiAl crystallite size. This improvement is related to the Ti–Mn–Co–Fe–Cr metal alloys' pinning effect, which was made feasible by the tubular mixing procedure with an extreme homogeneity. Thus, the improved dislocation density, reduced dislocation movement, and suppressed grain growth were all achieved by the refined alloys. In addition, as shown in Fig. 8, an increase in microstrain distribution was noted as a result of the increasing Ti–Mn–Co–Cr weight constituents. Unalloyed NiAl had the lowest microstrain distribution, measuring around 0.00187 ± 0.001, whereas Ni_25_Al_25_Ti_8_Mn_8_Co_15_Fe_14_Cr_5_ had the highest microstrain, measuring 0.0449 ± 0.003. These changes in microstrain and crystallite size can be related to the mixing procedure, the sintering conditions, the impact of the alloying metals, and the good plastic deformation [62, 76–78].

### Physical properties analysis of Ni–Al–Ti–Mn–Co–Fe–Cr sintered HEA

**Fig. 9:**
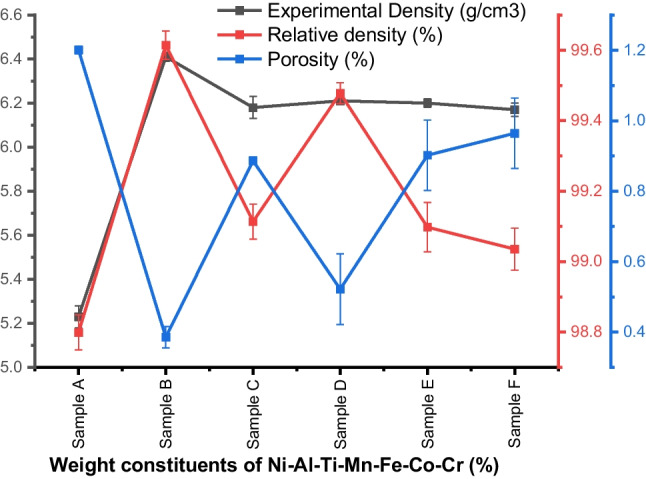
Density, relative density, and porosity of sintered Ni–Al–Ti–Mn–Co–Fe–Cr HEA at different weight compositions: **A** Ni_50_Al_50_, **B** Ni_14.286_Al_14.286_Ti_14.286_Mn_14.286_Co_14.286_Fe_14.286_Cr_14.286_, **C** Ni_25_Al_25_Ti_8_Mn_8_Co_14_Fe_15_Cr_5_, **D** Ni_25_Al_25_Ti_8_Mn_8_Co_15_Fe_14_Cr_5_, **E** Ni_25_Al_25_Ti_8_Mn_9_Co_14_Fe_14_Cr_5_, and **F** Ni_25_Al_25_Ti_9_Mn_8_Co_14_Fe_14_Cr_5_

The density, relative density, and percentage porosity variations of the sintered Ni–Al–Ti–Mn–Co–Fe–Cr HEA are depicted in Fig. 9 and Table 6, respectively. From the result obtained, it could be noted that the varied weight percentage of the constituent’s alloys Ti–Mn–Co–Fe–Cr in the master allow NiAl influence the relative density. The master alloy, Ni_50_Al_50_, has lower sintered density compared to the developed HEA, this could be credited to the primary density of the alloying elements. However, from the result of the unequal atomic HEA, Ni_25_Al_25_Ti_8_Mn_8_Co_15_Fe_14_Cr_5_ is seen to be denser compared to others, this could be as a result of the percentage weight of cobalt which is higher. Another explanation for the decline in densification is that the precipitated BCC B2 eutectic phase did not dissolve completely along the grain boundaries, leading to partial wettability and minor clustering or agglomeration [62]. A decrease in relative density and an increase in porosity characterize the master alloy. The clustering or agglomeration that happens at the borders of the alloying metals may be the cause [62, 79, 80]. The presence of high porosity in a material results in material failure such as fractures and cracks. Nevertheless, for a material failure to occur, the percentage porosity must be above the critical point of 4% [81]. For the developed HEA, the percentage porosity was lower than 1%, which makes the material suitable for use.

**Table 6:** Physical properties of the sintered Ni–Al–Ti–Mn–Co–Fe–Cr HEA at different weight compositions

Sample	Experimental density (g/cm^3^)	Relative density (%)	Porosity (%)
Ni_50_Al_50_	5.23 ± 0.047	98.799 ± 0.048	1.20 ± 0.011
Ni_14.286_Al_14.286_Ti_14.286_Mn_14.286_Co_14.286_Fe_14.286_Cr_14.286_	6.41 ± 0.017	99.614 ± 0.036	0.39 ± 0.028
Ni_25_Al_25_Ti_8_Mn_8_Co_14_Fe_15_Cr_5_	6.18 ± 0.046	99.114 ± 0.049	0.89 ± 0.014
Ni_25_Al_25_Ti_8_Mn_8_Co_15_Fe_14_Cr_5_	6.21 ± 0.01	99.478 ± 0.027	0.52 ± 0.1
Ni_25_Al_25_Ti_8_Mn_9_Co_14_Fe_14_Cr_5_	6.2 ± 0.02	99.097 ± 0.072	0.90 ± 0.1
Ni_25_Al_25_Ti_9_Mn_8_Co_14_Fe_14_Cr_5_	6.17 ± 0.03	99.035 ± 0.061	0.96 ± 0.1

### Mechanical properties analysis of sintered Ni–Al–Ti–Mn–Co–Fe–Cr HEA

#### Microhardness behavior of sintered Ni–Al–Ti–Mn–Co–Fe–Cr HEA

**Fig. 10:**
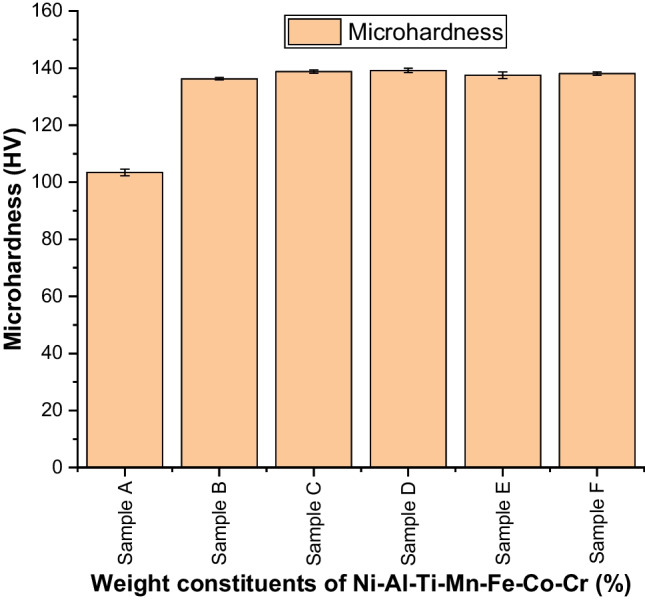
Microhardness behavior of the of sintered Ni–Al–Ti–Mn–Co–Fe–Cr HEA at different weight compositions: **A** Ni_50_Al_50_, **B** Ni_14.286_Al_14.286_Ti_14.286_Mn_14.286_Co_14.286_Fe_14.286_Cr_14.286_, **C** Ni_25_Al_25_Ti_8_Mn_8_Co_14_Fe_15_Cr_5_, **D** Ni_25_Al_25_Ti_8_Mn_8_Co_15_Fe_14_Cr_5_, **E** Ni_25_Al_25_Ti_8_Mn_9_Co_14_Fe_14_Cr_5_, and **F** Ni_25_Al_25_Ti_9_Mn_8_Co_14_Fe_14_Cr_5_

The sintered HEAs behavior with respect to microhardness is depicted in Fig. 10. The outcome showed that, in comparison with unalloyed NiAl, which had a hardness of 103.5 ± 1.2 HV, the developed HEA had a maximum hardness of 139.2 ± 0.8 HV and a percentage enhancement of roughly 17.6% (Table 7). Ni_25_Al_25_Ti_8_Mn_8_Co_15_Fe_14_Cr_5_ with the highest weight cobalt composition was able to attain this enhanced hardness (18.08 wt.%). The result demonstrates how grain size affects the mechanical properties of the developed HEA. Figure 10 shows a close range of the hardness value, but from the result from Table 7 clearly distinguishes the hardness value not been constant. However, the results of samples C–F are close, which is a reflection of the grain size. The marginal differences in the composition are close, too; this may also account for the close hardness observed. According to this result, the continuous matrix alloys' interphase's particle size and weight constituents have increased the alloy's ability to support a load and reduced its susceptibility to deformation. Strong adhesive bonding at the interface of the alloying element caused by the greater surface area of the nano-inclusions was what gave the material its outstanding hardness [62]. The combined effect of electromagnetic energy and green compact of powders creates a more dense and uniform material, which was made possible by the spark plasma sintering at a crucially shorter sintering time [76, 82]. The precipitation of the coarse NiAl eutectic phase along the grain boundaries was caused to dissolve during the sintering process by the nucleation of micro-sized Ti–Mn–Co–Fe–Cr particles in the solid solution of Ni–Al–Ti–Mn–Co–Fe–Cr and form homogenized, evenly distributed fine particles at the borders. The strengthening of the HEA was made possible by this phase of refinement [62, 83, 84]. Thus, the increased grain size brought on by the populated coarse NiAl eutectic phase (BCC B2 phase) accounts for the unalloyed NiAl's reduced hardness.

**Table 7:** Mechanical properties of the sintered Ni–Al–Ti–Mn–Co–Fe–Cr HEA at different weight compositions

Sample	Elastic modulus (GPa)	Nanohardness (GPa)	Microhardness (HV)	Penetration depth (nm)	Elastic strain (*H*/*E*_r_)	Yield pressure (*H*^3^/$E_{{\text{r}}}^{2}$)	Elastic recovery (*W*_e_/*W*_t_)	Plasticity index (*W*_p_/*W*_t_)
A	180 ± 5	10.2 ± 0.4	103.5 ± 1.2	703.6542	0.0567 ± 0.0061	0.0328 ± 0.0051	0.415 ± 0.007	0.585 ± 0.068
B	198.8 ± 1	15.8 ± 0.4	136.3 ± 0.5	598.2391	0.0795 ± 0.0073	0.0998 ± 0.0075	0.484 ± 0.013	0.516 ± 0.039
C	206.34 ± 2.8	18.35 ± 0.9	138.8 ± 0.6	562.8766	0.0889 ± 0.0093	0.145 ± 0.0077	0.5467 ± 0.028	0.454 ± 0.019
D	207.5 ± 1.6	18.8 ± 0.4	139.2 ± 0.8	520.5452	0.0906 ± 0.0027	0.154 ± 0.0055	0.556 ± 0.035	0.444 ± 0.039
E	204.12 ± 0.6	18.31 ± 0.3	137.5 ± 0.2	542.4921	0.0897 ± 0.0064	0.147 ± 0.077	0.517 ± 0.005	0.482 ± 0.024
F	203.7 ± 0.8	18.28 ± 0.02	138.05 ± 0.6	555.6471	0.0897 ± 0.0059	0.147 ± 0.0081	0.520 ± 0.028	0.479 ± 0.036

#### Nanoindentation of sintered Ni–Al–Ti–Mn–Co–Fe–Cr HEA

**Fig. 11:**
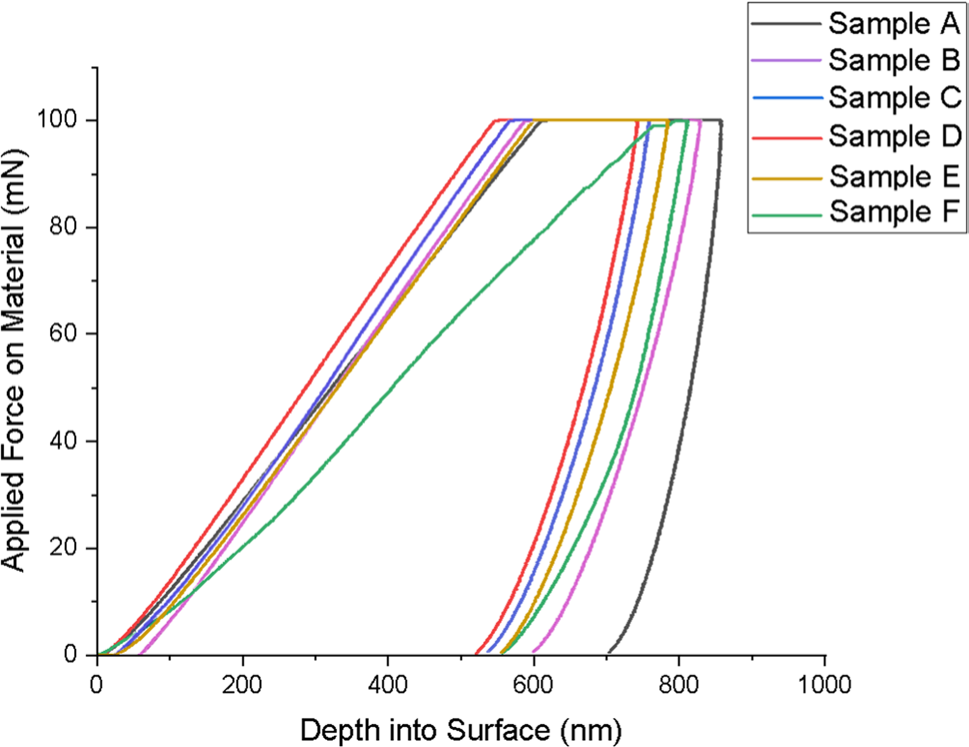
Nanoindentation load–displacement curve of sintered Ni–Al–Ti–Mn–Co–Fe–Cr HEA: **A** Ni_50_Al_50_, **B** Ni_14.286_Al_14.286_Ti_14.286_Mn_14.286_Co_14.286_Fe_14.286_Cr_14.286_, **C** Ni_25_Al_25_Ti_8_Mn_8_Co_14_Fe_15_Cr_5_, **D** Ni_25_Al_25_Ti_8_Mn_8_Co_15_Fe_14_Cr_5_, **E** Ni_25_Al_25_Ti_8_Mn_9_Co_14_Fe_14_Cr_5_, and **F** Ni_25_Al_25_Ti_9_Mn_8_Co_14_Fe_14_Cr_5_

The sintered HEAs nanoindentation load–displacement depth curve is shown in Fig. 11 for various weight constituents. Excellent elastic–plastic characteristics of materials undergoing deformation may be easily seen from the plot. There is no physical evidence of a pop-in effect noticed from the load–displacement curve. The presence of pop-in translates to material defect or deformation with a consequential effect on the material strength. This frequently happens when the diamond indenter makes contact with the materials being tested, causing dislocation movement and crack propagation [85–88]. The absence of pop-in could be attributed to the shielding of possible dislocation activities and crack initiation that was halted as a result of the presence of nanoparticles. In contrast with the unalloyed NiAl alloy, the developed HEA strength was thereby improved against high indenter penetration. It is of essence to note that the more the penetration depth, the more pop-in effect [62, 87]. Table 7 shows the nanoindentation outcome.

**Fig. 12:**
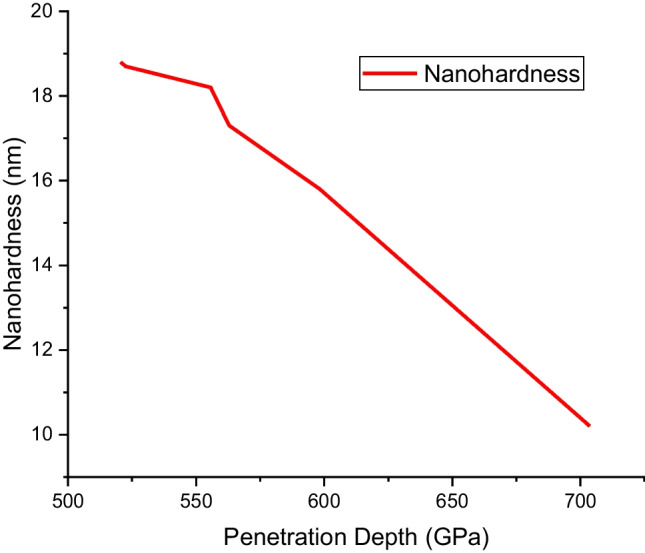
Nanohardness vs penetration depth curve of the sintered Ni–Al–Ti–Mn–Co–Fe–Cr HEA: **A** Ni_50_Al_50_, **B** Ni_14.286_Al_14.286_Ti_14.286_Mn_14.286_Co_14.286_Fe_14.286_Cr_14.286_, **C** Ni_25_Al_25_Ti_8_Mn_8_Co_14_Fe_15_Cr_5_, **D** Ni_25_Al_25_Ti_8_Mn_8_Co_15_Fe_14_Cr_5_, **E** Ni_25_Al_25_Ti_8_Mn_9_Co_14_Fe_14_Cr_5_, and **F** Ni_25_Al_25_Ti_9_Mn_8_Co_14_Fe_14_Cr_5_

The unalloyed NiAl alloy shows a higher penetration depth of 703.65 nm followed by the equal atomic (Ni_14.286_Al_14.286_Ti_14.286_Mn_14.286_Co_14.286_Fe_14.286_Cr_14.286_) at a depth of 598.24 nm, and the least penetration depth of 520.54 nm was observed for Ni_25_Al_25_Ti_8_Mn_8_Co_15_Fe_14_Cr_5_. The reduction in penetration was achievable by the inclusion of Ti–Mn–Co–Fe–Cr materials. The nanohardness varies inversely with the penetration depth. As the nanohardness increases, the penetration depth reduces as shown in Fig. 12.

**Fig. 13:**
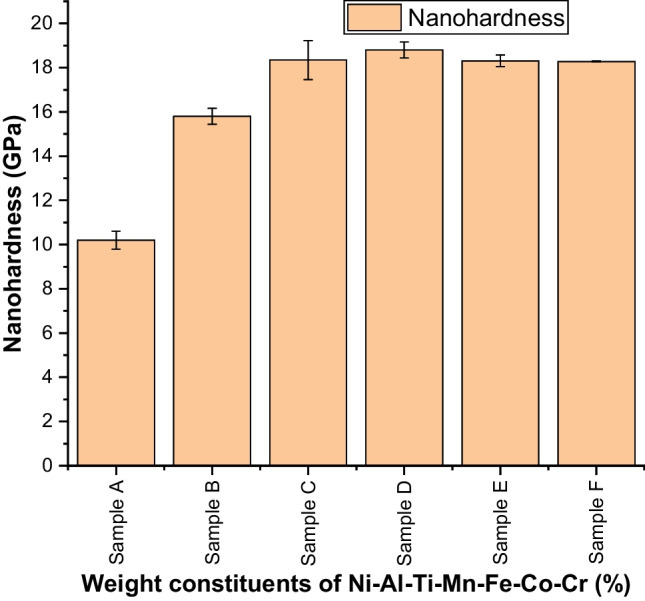
Nanohardness behavior of sintered Ni–Al–Ti–Mn–Co–Fe–Cr HEA: **A** Ni_50_Al_50_, **B** Ni_14.286_Al_14.286_Ti_14.286_Mn_14.286_Co_14.286_Fe_14.286_Cr_14.286_, **C** Ni_25_Al_25_Ti_8_Mn_8_Co_14_Fe_15_Cr_5_, **D** Ni_25_Al_25_Ti_8_Mn_8_Co_15_Fe_14_Cr_5_, **E** Ni_25_Al_25_Ti_8_Mn_9_Co_14_Fe_14_Cr_5_, and **F** Ni_25_Al_25_Ti_9_Mn_8_Co_14_Fe_14_Cr_5_

**Fig. 14:**
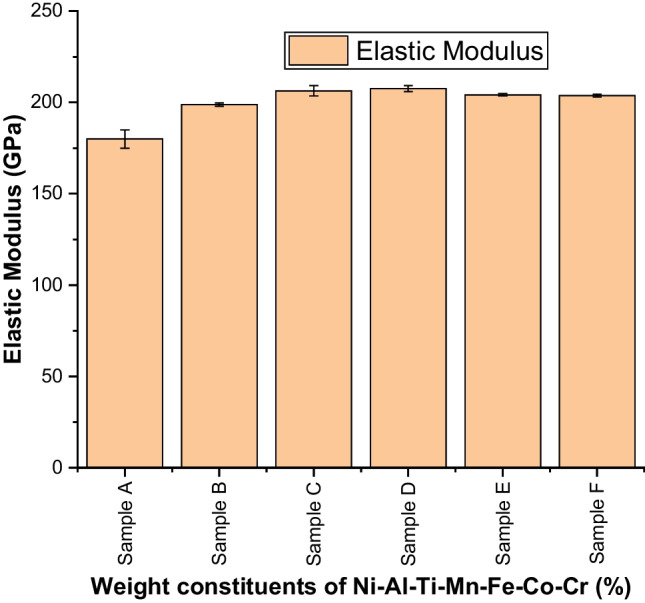
Elastic modulus of sintered Ni–Al–Ti–Mn–Co–Fe–Cr HEA: **A** Ni_50_Al_50_, **B** Ni_14.286_Al_14.286_Ti_14.286_Mn_14.286_Co_14.286_Fe_14.286_Cr_14.286_, **C** Ni_25_Al_25_Ti_8_Mn_8_Co_14_Fe_15_Cr_5_, **D** Ni_25_Al_25_Ti_8_Mn_8_Co_15_Fe_14_Cr_5_, **E** Ni_25_Al_25_Ti_8_Mn_9_Co_14_Fe_14_Cr_5_, and **F** Ni_25_Al_25_Ti_9_Mn_8_Co_14_Fe_14_Cr_5_

Figures 13 and 14 show an incredible increase in the nanohardness and elastic modulus plots, as a result of reinforcing Ti–Mn–Co–Fe–Cr materials. As observed in Fig. 10, there is a similar trend in Fig. 13, with Ni_25_Al_25_Ti_8_Mn_8_Co_15_Fe_14_Cr_5_ exhibiting a higher value of nanohardness of 18.8 ± 0.36 GPa compared to 10.2 ± 0.4 GPa from the unalloyed NiAl alloy. This result is a function of particle size, and weight constituents which significantly enhanced the load bearing capacity, work hardening, and reduces the dislocation movement. As a result, the material's strength and elastic modulus improved while the penetration depth decreased.

**Fig. 15:**
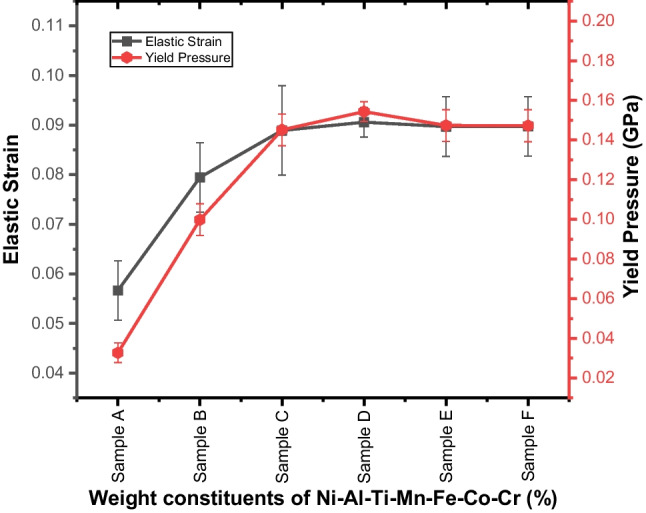
Elastic strain (H/E_r_) and yield pressure (H^3^ /E^2^_r_) of sintered Ni–Al–Ti–Mn–Co–Fe–Cr HEA: **A** Ni_50_Al_50_, **B** Ni_14.286_Al_14.286_Ti_14.286_Mn_14.286_Co_14.286_Fe_14.286_Cr_14.286_, **C** Ni_25_Al_25_Ti_8_Mn_8_Co_14_Fe_15_Cr_5_, **D** Ni_25_Al_25_Ti_8_Mn_8_Co_15_Fe_14_Cr_5_, **E** Ni_25_Al_25_Ti_8_Mn_9_Co_14_Fe_14_Cr_5_, and **F** Ni_25_Al_25_Ti_9_Mn_8_Co_14_Fe_14_Cr_5_

Figure 15 displays the materials' elastic strain to failure and yield pressure, which were estimated using the correlation between hardness and elastic modulus. The plot indicates how much the material can withstand plastic deformation. The unalloyed NiAl alloy exhibited a low resistance to plastic deformation due to its low yield pressure and least elastic strain to failure of 0.0567 ± 0.006 and 0.0328 ± 0.005 GPa, respectively. However, the Ti–Mn–Co–Fe–Cr addition improves the resistance to deformation, and the Ni_25_Al_25_Ti_8_Mn_8_Co_15_Fe_14_Cr_5_ was able to achieve a higher resistance with values of 0.0906 ± 0.003 and 0.1543 ± 0.005 GPa, respectively. This is an indication that the developed HEA does not only possess an excellent mechanical property but also an enhanced bonding link structure. Consequently, by preventing the dislocation movement, this lessens the likelihood that the materials may fail in service [62, 76, 82].

**Fig. 16:**
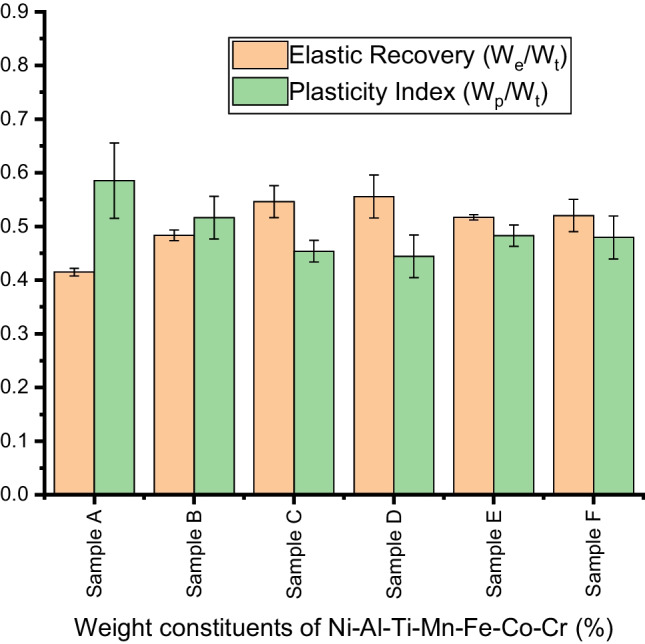
Elastic strain (*H*/*E*_r_) and yield pressure (*H*^3^/$E_{{\text{r}}}^{2}$) of sintered Ni–Al–Ti–Mn–Co–Fe–Cr HEA: **A** Ni_50_Al_50_, **B** Ni_14.286_Al_14.286_Ti_14.286_Mn_14.286_Co_14.286_Fe_14.286_Cr_14.286_, **C** Ni_25_Al_25_Ti_8_Mn_8_Co_14_Fe_15_Cr_5_, **D** Ni_25_Al_25_Ti_8_Mn_8_Co_15_Fe_14_Cr_5_, **E** Ni_25_Al_25_Ti_8_Mn_9_Co_14_Fe_14_Cr_5_, and **F** Ni_25_Al_25_Ti_9_Mn_8_Co_14_Fe_14_Cr_5_

The elastic recovery and plasticity index of the fabricated Ni–Al–Ti–Mn–Co–Fe–Cr HEA are shown in Fig. 16. The alloyed NiAl shows a higher recovery index compared to the unalloyed NiAl alloy. Ni_25_Al_25_Ti_8_Mn_8_Co_15_Fe_14_Cr_5_ has a higher recovery index of 0.556 ± 0.04 while NiAl has the least recovery index of 0.41 ± 0.007. The manufactured HEAs plasticity index, however, was shown to decrease with changes in the weight percent of its constituents, with the unalloyed NiAl alloy exhibiting the highest plasticity index. As a result, it appears that NiAl experiences a severe plastic deformation with little stiffness, which may have facilitated the elastic recovery. The elastic recovery index of materials indicated their resistance to impact loading by assessing the amount of energy released after being loaded, whereas the plasticity index denotes the inherent plasticity of the basic material [89]. As a result, the findings of this innovative study with high elastic recovery demonstrated that alloyed NiAl emits little energy while maintaining high elastic energy, with Ni_25_Al_25_Ti_8_Mn_8_Co_15_Fe_14_Cr_5_ exhibiting the maximum energy.

## Novelty of the study

The novelty of this research is established in the HEA developed via advanced manufacturing route. These material combinations have not been investigated before in procuring solutions to identified problems faced by NiAl alloy. The development of novel NiAl-based HEA is suitable for multifunctional performance in advanced engineering application. Provision of relevant research information on the process–structure–property relationship of NiAl-based HEAs might be useful for advanced materials engineering research.

## Conclusion

This innovative work successfully incorporated Ti–Mn–Co–Fe–Cr material to NiAl using SPS in order to improve its mechanical properties. After the exploratory experiment, the finding can be summarized as follows:

1. The developed NiAl-based HEA was refined to attain a reduced crystallite size, minimal grain size, and maximum microstrain by the inclusion of Ti–Mn–Co–Fe–Cr nanomaterial through mechanical alloying. After sintering, Ni_25_Al_25_Ti_8_Mn_8_Co_15_Fe_14_Cr_5_ had crystallite size, grain size, and microstrain values of 2.081 ± 0.2 nm, 2.36 ± 0.4 μm, and 0.0449 ± 0.004 compared to unalloyed Ni_50_Al_50_, which had 8.22 ± 0.18 nm, 8.26 ± 0.4 μm, and 0.00188 ± 0.0005, respectively, which exhibit larger grains and lower microstrain. This intricate structure aids in restricting dislocation movement.
2. The load–displacement curve's penetration depth was observed to be declining, which is consistent with a increase in the sintered alloys' nanohardness trend.
3. The Ni_25_Al_25_Ti_8_Mn_8_Co_15_Fe_14_Cr_5_ has the highest microhardness, nanohardness, and elastic modulus of 139.2 ± 0.8 HV, 18.8 ± 0.36 GPa, and 207.5 ± 1.6 GPa, respectively. While Ni_50_Al_50_ has the least.
4. The unalloyed NiAl is predominantly made up of BCC_B2 phase, the equal atomic HEA has three phases, namely, FCC, BCC, and SIGMA while the non-equal atomic HEAs are made up of BCC, SIGMA, and HEUSLER phases.

## Data Availability

The datasets generated during and/or analyzed during the current study are available from the corresponding author on reasonable request.
